# Polo-like kinase 1 as target for cancer therapy

**DOI:** 10.1186/2162-3619-1-38

**Published:** 2012-12-10

**Authors:** Lily Weiß, Thomas Efferth

**Affiliations:** 1 Department of Pharmaceutical Biology, Institute of Pharmacy and Biochemistry, Johannes Gutenberg University, Staudinger Weg 5, 55128, Mainz, Germany

**Keywords:** Small molecules, Kinase, Prognostic marker, Targeted chemotherapy

## Abstract

Polo-like kinase 1 (Plk1) is an interesting molecule both as a biomarker and as a target for highly specific cancer therapy for several reasons. Firstly, it is over-expressed in many cancers and can serve as a biomarker to monitor treatment efficacy of Plk1 inhibitors. Furthermore, the Plk1 enzyme is expressed only in dividing cells and is a major regulator of the cell cycle. It controls entry into mitosis and regulates the spindle checkpoint. The expression of Plk1 in normal cells is not nearly as strong as that in cancer cells, which makes Plk1 a discriminating tartget for the development of cancer-specific small molecule drugs. RNA interference experiments *in vitro* and *in vivo* have indicated that downregulation of Plk1 expression represents an attractive concept for cancer therapy. Over the years, a number of Plk1 inhibitors have been discovered. Many of these inhibitors are substances that compete with ATP for the substrate binding site. The ATP-competitive inhibitor BI 6727 is currently being clinically tested in cancer patients. Another drug in development, poloxin, is the first Polo-box domain inhibitor of Plk1. This compound is a derivative of the natural product, thymoquinone, derived from *Nigella sativa*. A novel and promising strategy is to synthesize bifunctional inhibitors that combine the high binding affinity of ATP inhibitors with the specificity of competitive inhibitors.

## Introduction

Polo-like kinase 1 (Plk1) plays a critical role in cell division and represents a promising target for cancer therapy. Several small molecule inhibitors of polo-like kinase 1 have been described. In the present review, we focus on the biology of the Plk1 enzyme and the significance of small molecule inhibitors as novel candidates for cancer therapy. Polo-like kinases are serine-threonine protein kinases. There are four different polo-like kinases (Plk 1-4), of which Plk1 is the best studied. All Plks consist of an amino-terminal catalytic kinase domain, which is responsible for ATP-binding and enzyme activation, and a carboxy-terminal polo box domain (PBD). The PBD is involved in phosphopeptide binding and consists of either one (in Plk 4) or two sequence motifs (in Plk 1, Plk 2, and Plk 3) which each consist of 80 residues [1]. Phosphopeptides bind to the two motif-PBDs at the consensus sequence S-pS/pT-P/X [2]. The PBDs of the four Plks are similar, but not identical and have different binding affinities. Plk1 and Plk 2 share the most similar PBDs, whereas Plk4’s PBD is most dissimilar. Because the Plk4 PBD consists of only one motif, it does not form a binding pocket as the other Plks do [1,3]. Plk1 consists of 603 amino acids [4].

## The molecular biology of polo-like kinase 1

### Structure of polo-like kinases

Plks possess a conserved catalytic kinase domain, which consists of two lobes, each composed of a polypeptide chain. A hinge region connects these two lobes allowing their rotation. ATP molecules can bind at the gap between the two polypeptide chains. The binding pocket structure is highly conserved among all Plks. In general, all protein kinases reveal similar ATP-binding pockets, which may pose problems in developing highly specific kinase inhibitors [1,3].

### Function of Plk1

Protein kinases are important for a multitude of cellular signal transduction reactions and are, therefore, subject to strong regulation. They can be activated or inactated by interaction with other proteins, peptides, or small molecules or by translocation in the cell. Protein kinases modify the activity of other proteins by phosphorylation. In a reversible process, the kinase catalyzes the transfer of the γ-phosphate from a donor molecule (ATP) to alcoholic (serine, threonine) or phenolic (tyrosine) amino acids, which bear a free hydroxyl group. Plk1 phosphorylates serine and threonine [5,6].

During mitosis, Plk1 binds and phosphorylates multiple proteins and hence plays a crucial role in cell proliferation. It is therefore not surprising that Plk1 is only expressed in dividing cells. During cell cycle progression, fluctuations of Plk1 expression and activity can be detected. The highest Plk1 levels can be observed during the late G2 and M phases of the cell cycle. Furthermore, Plk1’s cellular localization changes in the course of mitosis [7].

Plk1 is involved in the activation of cyclin B1 and Cdc25C, and thereby triggers entry of the cell into mitosis. Furthermore, Plk1 plays a role in the maturation of centromers, the formation and maintenance of the bipolar spindle, the spindle checkpoint, exit from mitosis, and cytokinesis. During interphase and prophase, Plk1 is localized to the centrosome. In prometaphase and metaphase, it can be detected at the kinetochore and at the spindle poles. During anaphase and telophase, Plk1 is displaced to the central spindles [7].

In addition to promoting proliferation of healthy cells, Plk1 also plays an important role in cancer development. If Plk1 harbors mutations and is overexpressed, the mitotic cell cycle checkpoint is affected. Several cell cycle checkpoints maintain genomic stability in normal cells, including the G1, G2/M- and spindle checkpoints. Upon DNA damage or other cellular injury, the cell cycle is normaly arrested to allow for either damage repair or for induction of apoptosis, if the extent of damage exceeds that which the cell can repair. In cancer cells, cell cycle arrest can be overridden due to defective checkpoints. Frequently, checkpoints are bypassed by overexpression of mitotic kinases involved in the control of the checkpoints. As a consequence, proper cell division is no longer guaranteed. Faulty chromosomal alignment and distribution can occur, leading to aneuploidy. This is a typical phenomenon in cancer cells. After DNA damage, Plk1 activity allows cells in G2/M arrest to re-enter mitosis by activating cyclin-dependent kinase 1 (Cdk1).

## Prognostic value of Plk1 in clinical tumors

Compared to normal tissues, Plk1 is overexpressed in many cancers, *e.g.* breast cancer, ovarian carcinoma colorectal carcinoma, uterine cancer, skin cancer, stomach cancer, and others. To estimate whether or not PLK1 expression is of prognostic value, we have summarized the literature on this topic in Table 1.

**Table 1:** Role of Plk1 as prognostic marker in clinical tumors

**Tumor type**	**Number of tumors**	**Method**	**Result**	**Reference**
colorectal cancer	78	immunohistochemistry	expression in tumors higher than in normal tissues	[8]
			significant correlations with invasion and Duke’s stage	
advanced rectal cancer	76	immunohistochemistry	significant correlations with tumor regression	[9]
	20	mRNA microarray	and long-term clinical outcome	
colorectal carcinoma	56	immunohistochemistry	expression in tumors higher than in normal tissues	[10]
			significant correlations with Duke’s stage, tumor size,	
			invasion, and lymph node metastasis	
gastric adenocarcinoma	208	immunohistochemistry	expression in tumors higher than in normal tissues	[11]
		RT-PCR		
gastric adenocarcinoma	160	immunohistochemistry	prognostic factor for poor survival time	[12]
		RT-PCR		
gastric adenocarcinoma	135	immunohistochemistry	expression in tumors higher than in normal tissues	[13]
			significant correlations with stage, nodal status and diffuse	
			growth pattern, no correlation to lymph node metastasis	
hepatocellular carcinoma	135	immunohistochemistry	expression in tumors higher than in normal tissues,	[14]
	111	RT-PCR	significant correlations to venous invasion, tumor nodules	
			and Edmondson grade, prognostic factor for poor survival time	
hepatoblastoma	74	RT-PCR	expression in tumors higher than in normal tissues	[15]
non-small cell lung	100	immunohistochemistry	expression in tumors higher than in normal tissues	[16]
cancer			significant correlations with stage, grade and lymph	
			node metastasis	
melanoma	36		significant correlation with metastasis	
astrocytic tumors		immunohistochemistry	significant correlation with grade of anaplasia	[17]
non-Hodgkin lymphoma	66	immunohistochemistry	prognostic factor for poor survival time	[18]
non-Hodgkin lymphoma	118	immunohistochemistry	significant correlations with grade,	
			prognostic factor for poor survival time	
cutaneous T-cell	49	immunohistochemistry	expression in tumors higher than in patch and	[19]
lymphoma			plaque-stage lesions	
muliple myeloma	188	immunohistochemistry	prognostic factor for poor survival time	[20]
breast carcinoma	135	immunohistochemistry	expression in tumors higher than in normal tissues	[21]
			no correlation with 10 year survival time	
breast carcinoma	3093	immunohistochemistry	no correlation with 10 year survival time	[22]
ovarian tumors	17	immunohistochemistry	significant correlation with grade	[23]
ovarian tumors	107	immunohistochemistry	expression in high malignant tumors than in low	[24]
			malignant tumors, prognostic factor for poor survival time	
endometrial tumors	20	immunohistochemistry	significant correlation with grade	[25]
cervical carcinoma	36	immunohistochemistry	significant correlation with tumor size	[26]

Whereas most studies used immunohistochemistry to analyze Plk1 expression, a few studies also used RT-PCR to measure mRNA of Plk1 in human tumor samples. This difference is of interest becuase it has long been a matter of discussion which methods are most suitable for clinical routine diagnosis of molecular markers [27]. Despite the limitations of immunohistochemical detection of protein expression, this technique is often used for practical reasons to investigate formalin-fixed and paraffin-embedded tumor biopsies, which are the most frequently used samples in pathology laboratories.

Table 1 demonstrates that Plk1 expression correlates with clinicopathological parameters such as stage, grade, metastasis, and survival time of patients. This indicates that Plk1 may serve as valuable marker for prognosis of treatment outcome and survival time.

## Plk1 inhibitors

### Approaches for the design of specific Plk1 inhibitors

Protein kinases have garnered great interest as therapeutic targets because they are involved in numerous diseases including inflammatory, neurological, and neoplastic diseases [28]. In malignant cells, signal transduction is often dysregulated due to mutated-activated protein kinases. The elucidation of the structure and function of cell cycle regulators is critically important for the development of target-specific therapeutic inhibitors. In addition to being therapeutically relevant, kinase inhibitors may also serve as biochemical tools to explore signal transduction processes in experimental models.

Since Plk1 is overexpressed in many cancers, it represents an interesting target molecule for the development of specific inhibitors to selectively treat cancer while avoiding toxicity towards normal tissues [28,29].

#### Inhibition of Plk1 gene expression

There are several possibilities for suppressing Plk1 expression at the translation level. One mechanism with therapeutic implications is the naturally occurring process of RNA interference (RNAi), which is triggered by double stranded RNA. RNAi leads to suppressed expression of genes with complementary sequences. RNA fragments of about 21 nucleotides, called small interfering RNAs (siRNA), can be enzymatically cleaved into single strands of RNA by an enzyme complex known as RNA-induced silencing complex (RISC). The single-stranded RNA fragments bind to complementary mRNA resulting in its degradation. For the specific inhibition of Plk1, siRNAs complementary to *PLK1* mRNA have been described that degrade *PLK1* mRNA and downregulate Plk1 protein levels [28].

Furthermore, *PLK1* gene expression can be downregulated by single-stranded DNA antisense oligonucleotides. These antisense oligos bind to the complementary mRNA and thereby prevent translation of *PLK1*[28].

Inhibition of Plk1 by RNA interference in cancer cells *in vitro* resulted in mitotic arrest and subsequent apoptosis [1]. These studies showed that cancer cells apparently have no back-up mechanism to bypass Plk1 inhibition. This makes Plk1 a promising target for small molecule drug development, as Plk1 RNAi is difficult to acheive *in vivo*. Nevertheless, Strebhardt et al. [3] were able to inhibit *PLK1* expression by RNAi both *in vitro* and *in vivo,* and demonstrated a specific killing of cancer cells while normal cells survived. The reasons for this result are still not completely understood. It is thought that *PLK1* siRNA might not only partially inhibit Plk, and that the residual enzyme activity is sufficient for normal cells to survive. Another explanation could be that normal cells activate alternative pathways to respond to siRNA-mediated challenge, allowing them to survive. This may be a siRNA-specific phenomenon, since chemical small molecules such as BI 2536 or Poloxin inhibited both proliferating normal and malignant cells in a consistent manner *in vitro*[2]. Overall, the development of therapeutic siRNAs to inhibit PLK1 gene expression level may be conceivable.

#### Functional Plk1 inhibitors

A detailed knowledge of the exact structural configuration and functional operation of Plk1 is a prerequisite for the synthesis of highly specific polo-like kinase 1 inhibitors. In this context, the phosphorylation reactions play a crucial role, because this is the starting point for the design of specific inhibitors. Different inhibitors can be distinguished depending on their mode of action: inhibitors can compete with either ATP or the substrate or with both ATP and the substrate of Plk1 [5].

Because the ATP binding pocket represents a classic target for the synthesis of kinase inhibitors, the first designed Plk1 inhibitors were ATP analogues that block the ATP binding pocket and thereby prevent access to ATP. For the design of novel inhibitor scaffolds, computer-based methods such as pharmacophore modeling and virtual screening are now used. After identification of candidate compounds in large chemical libraries, wet lab experiments must be used to verify the binding between the compounds and Plk1. Chemical scaffolds may serve as lead compounds which can be subsequently optimized by derivatization to acheive more specific binding to Plk1. In this context, the Plk1 amino acids Arg136 and Leu13 are essential for inhibitor binding, as in the case of BI 2536 [3]. Nevertheless, inhibitors designed to bind to Plk1 are also capable of binding non-specifically to other protein kinases, because all protein kinases share a similar amino acid sequence and highly conserved structure in their ATP-binding pocket. The probability is high that ATP-competitive Plk1 inhibitors will also bind to other members of the PLK family (with the exception of Plk4, which is dissimilar to the other Plks). Therefore, it is a non-trivial task to develop Plk1-specific inhibitors. For example, the Plk1 inhibitors PHA-680626 and BI 2536 do not sufficiently distinguish between Plk1, Plk2, and Plk3 and interact with all three. Second generation inhibitors such as ZK-thiazolidinone, NMS-P937, GSK461364, or BI 2767 are believed to be more specific [3].

To increase the specificity of Plk1 inhibition, substances binding to the PBD and competing with natural Plk1 substrates have been developed. These compounds are able to block protein-protein interactions and consist of modified peptides, which contain consensus sequences for the corresponding substrates. As proof-of-principle, it was shown for protein kinase A (PKA) that peptide analogs with longer consensus sequences have a greater inhibitory effect than peptide analogs with shorter substrate consensus sequences [30].

Plks are the only protein kinases that cotain PBDs. Therefore, it has been attempted to increase the specificity of inhibitors by using sequences complementary to the PBD. The PBD binds with high affinity peptides containing the consensus sequence S-pS/pT-P/X. The first substrate-competitive Plk1 inhibitor, which binds specifically to the PBD, was discovered using a recently developed fluorescence polarization assay. First, a fluorophore-coupled peptide binds with high affinity to the PBD of Plk1. Next, a chemical library of low molecular weight compounds was tested. These compounds interact with the Plk1 PBD and thus compete with the fluoropore-coupled peptide for access to the kinase, which can be measured by fluorescence polarization. The fluorescence polarization is higher when the labeled peptide is bound to the PBD of Plk1. The polarization is reduced upon displacement of the fluorescing peptide by an inhibitor. Applying this technology resulted in the discovery of synthetic poloxin as well as the natural product, thymoquinone [31]. Other inhibitors that bind to the PBD of Plk1 are purpurogallin and poloxipan [3].

As a next step to increase specificity, bifunctional inhibitors have been developed, which inhibit binding of both ATP and substrate via two domains connected by a linker. These compounds compete simultaneously with ATP and the peptide or protein substrate for their respective binding sites. The particular advantage of these bifunctional compounds is that they combine the high affinity of the ATP analogue, and the specificity of the substrate analogue. Ideally, such inhibitors should bind with high affinity to the target enzyme and simultaneously be able to discriminate between closely related specific protein kinases. The structure and the length of the linkage between the ATP and substrate analogues can greatly affect the efficiency of bifunctional inhibitors. As yet, bifunctional Plk1 inhibitors have not been developed [5,32].

Another type of inhibitor interacts with the kinase substrate to prevent phosphorylation. Since the inhibitor does not directly target the protein kinase itself in this approach, it is quite different from the other known kinase inhibitors. Such inhibitors for Plk1 are not yet known [5,33].

### The challenge to identify Plk1 inhibitors

The aim is to find substances that are able to inhibit Plk1-mediated phosphorylation of proteins by specifically and binding with high affinity to Plk1. Protein kinases are one of the largest protein families. The human proteome contains more than 2,000 different protein kinases of which Plk1 is just one [34]. This illustrates the magnitude of the task of identifying a Plk1 specific inhibitor; finding a compound that only inhibits one particular kinase is quite difficult, especially when there are so many structurally similar enzymes present in the cell.

Furthermore, ATP is used by all protein kinases as a donor molecule for γ-phosphate. The sequence and structure of the binding site is highly conserved, and therefore similar in all protein kinases, to enable ATP binding to the enzyme. This complicates the specific inhibition of Plk1. Another problem is the intracellular ATP concentrations, which are in the millimolar range. The Km values of protein kinases, however, are significantly lower, *i.e.* about one-thousandth of the intracellular ATP concentrations. Therefore, there is a saturation of ATP-binding protein kinases under physiological conditions. A novel ATP analogue must meet two criteria. It has to be specific to distinguish one from the other kinase and its binding affinity has to be high enough to displace the surplus ATP and the donor molecule [5].

Furthermore, protein kinases phosphorylate only certain amino acid residues. One system of kinase classification is based on the nature of the residue phosphorylated, *i.e.* some phosphorylate tyrosine and serine while others are solely threonine kinases. Therefore, the amino acid sequence of the substrate phosphorylation site is highly conserved. Protein kinases that phosphorylate the same amino acid residues show highly similar sequences, which complicates the design of specific binding inhibitors. The more closely related the proteins, the more similar are their binding sites. Since Plk1 through Plk3 (but not Plk4) contain a two-motif-PBD, they share very similar (but not identical) amino acid sequences for the substrate binding sites. The challenge is to design a non-phosphorylatable peptide analogue that specifically binds to the high affinity and substrate binding site of Plk1 [5].

## Examples of Plk1 inhibitors

### BI 6727

BI 6727 represents an ATP-competing kinase inhibitor belonging to a class called dihydropteridinones. It binds to the hinge region between the NH_2_-terminal end and the COOH-terminal end of the catalytic kinase domain. The binding takes place via two hydrogen bonds from the interior of the backbone of the NH- and carbonyl groups of Cys133. Thereby, BI 6727 occupies the ATP binding site of Plk1, which leads to a catalytical inactivation. BI 6727 stops cell division and the arrests the cell cycle in the G2/M phase, which is followed by apoptosis [29]*in vitro* and *in vivo*. This inhibitor was tested on 50 patients suffering from advanced or metastasized solid tumors in a clinical Phase I trial. Thrombocytopenia and neutropenia occurred as side effects [3]. It has been shown that BI 6727 inhibits Plk2 as well as Plk1, indicating that it is not the optimal candidate drug for specific Plk1 inhibition.

### Poloxin

Poloxin is a synthetical derivative of the phytochemical thymoquinone. It was the first small molecule identified to block Plk1 by specifically binding to the PBD. The identification was based on the screening of a chemical library by means of the above described fluorescence polarization assay [2]. Poloxin is the first non-peptide Plk1 inhibitor, and it prevents protein-protein interactions of Plk1’s two PBDS. The lead compound, thymoquinone, is a biologically active component of the seed oil of *Nigella sativa* volatile. Thymoquinone is known to exert anti-inflammatory, anti-oxidative and anti-neoplastic activities [3] and strongly inhibits Plk1, but also other protein kinases such as Plk2, Plk3, Chk2, Pin1 and STAT3. For this reason, the more specific derivative, poloxin, was synthesized. However, poloxin is less active than the natural lead compound. Therefore, its inhibitory effect towards the PBD of Plk1 was lower [31]. Poloxin reduced the localization of Plk1 at the kinetochore and the centrosomes, induced defects in chromosome arrangements, arrested cells in prometaphase, and induced apoptosis [2].

### GW843682X

The substance GW843682X was discovered during chemical optimization of a thiophene. Thiophenes were hypothesized to serve as chemical scaffolds for inhibitors because they are known to have a good inhibitory effect on Plk1. GW843682X is a competitive ATP inhibitor, which occupies the ATP binding site and inhibits the kinase activity. It arrested various cancer cell lines with aberrant Plk1 expression in the G2/M cell cycle phase and induced apoptosis. Furthermore, the anti-cancer effect of this compound was also demonstated *in vivo*. A clinical Phase I trial with patients suffering from advanced solid tumors was performed to determine the maximum tolerated dose and to perform pharmacokinetic analysis. The dose-limiting side effects of GW843682X include sepsis, pulmonary embolism, and neutropenia. GW843682X revealed 400-fold greater inhibitory activity towards Plk1 than towards Plk2 and Plk3 [3].

## Conclusion

PLK1 represents an exquisite prognostic marker and target for cancer therapy because of its strong expression in cancer but weak expression in normal tissues. A number of PLK1 inhibitors have been discovered during the past years, some of which are being tested in clinical trials. Once PLK1 inhibitors are clinically approved, they will represent an attractive novel strategy to improving treatment outcome.

## Competing interests

The authors declare that they have no competing interests.

## Authors’ contribution

LW has written the raw draft, TE has corrected and finalized the manuscript. Both authors read and approved the final manuscript.
